# Circulating Soluble Lectin-like Oxidized Low-Density Lipoprotein Receptor-1 (sLOX-1): A Diagnostic Indicator across the Spectrum of Acute Coronary Syndrome

**DOI:** 10.3390/jcm10235567

**Published:** 2021-11-26

**Authors:** Sandeep Kumar, Wahid Ali, Sridhar Mishra, Akshyaya Pradhan, Rishi Sethi, Rashmi Kushwaha, Uma Shankar Singh, Marco Alfonso Perrone

**Affiliations:** 1 Department of Pathology, King George’s Medical University, Lucknow 226003, India; sakdy27@gmail.com (S.K.); aliwahid78@gmail.com (W.A.); docrashmi27@yahoo.co.in (R.K.); ussinghjyotsna@yahoo.co.in (U.S.S.); 2 Department of Pathology, Dr. Ram Manohar Lohia Institute of Medical Sciences, Lucknow 226010, India; shridhar.mishra17@gmail.com; 3 Department of Cardiology, Lari Cardiology Centre, King George’s Medical University, Lucknow 226003, India; akshyaya33@gmail.com (A.P.); drrishisethi1@gmail.com (R.S.); 4 Department of Cardiology, University of Rome Tor Vergata, 00133 Rome, Italy

**Keywords:** coronary artery disease (CAD), low-density lipoprotein (LDL), acute coronary syndrome (ACS), soluble lectin-like oxidized low-density lipoprotein receptor-1 (sLOX-1)

## Abstract

Background: Cardiac troponin is the best marker to diagnose acute coronary syndrome (ACS). However, early diagnosis using markers for plaque instability may be of significance. Soluble lectin-like oxidized low-density lipoprotein receptor-1 (sLOX-1) plays an important role in the pathogenesis of atherosclerosis plaque rupture and may be a potential biomarker of coronary artery disease (CAD), including ACS. The current study aims to evaluate sLOX-1 levels in the sera of patients with ACS as an independent marker of CAD with other established diagnostic markers and assess its level before and after percutaneous intervention (PCI) in predicting the risk of future recurrence of ACS. Methods: Peripheral blood was obtained from a total of 160 patients, including patients who underwent coronary angiography (*n* = 18, group I), patients of stable CAD who underwent percutaneous intervention (*n* = 50, group II), patients of the acute coronary syndrome (*n* = 64, group III), and healthy controls (*n* = 28, group IV). A serum sLOX-1 concentration was measured by the enzyme-linked immunosorbent assay (ELISA). Results: The results obtained showed a statistically significant raised level of sLOX-1 in pre/post PCI patients of stable CAD/ACS with male preponderance. The area under the curve for sLOX-1 was 0.925 for cases that are discriminated from controls with sensitivity and specificity of 87.88 and 100%, respectively. SLOX-1 showed 100% sensitivity and specificity in the discrimination of the stable CAD that underwent PCI vs. control with an AUC of 1.00. The recurrence of coronary artery disease was observed in 9 out of 132 (6.8%) cases. The post-interventional sLOX-1 level was significantly different and higher in recurrent cases (*p* = 0.027) of ACS/CAD. Conclusions: sLOX-1 was a useful biomarker of stable CAD/ACS and has a potential in the risk prediction of a future recurrence of coronary artery disease.

## 1. Introduction

Cardiovascular disease remains a major cause of mortality and debility globally, of which coronary artery disease (CAD) accounts for the highest percentage [1], although the prognosis of CAD patients has improved with the use of percutaneous coronary intervention (PCI) and new drug therapies. However, CAD patients remain at a higher risk of adverse cardiovascular and cerebrovascular events [1]. Therefore, high-risk patients need to have a secondary prevention of adverse cardiovascular and cerebrovascular events. Newer emerging risk factors, particularly inflammatory and atherosclerotic burden markers, might identify those who are actually at a high risk and might benefit from a more aggressive risk reduction [2]. A comparative evaluation of these biomarkers is necessary to determine which are more suitable to be integrated into present strategies of therapeutic approaches. Many known markers have been studied so far [2].

Lectin-like oxidized low-density lipoprotein receptor-1 (LOX-1) is the receptor for atherogenic oxidized low-density lipoprotein (Ox-LDL). The pro-inflammatory stimuli and ox-LDL induce the expression of LOX-1, which affects the atherosclerotic progression and plaque vulnerability [3,4]. Soluble LOX-1 (sLOX-1) may be a useful predictive biochemical marker for CAD [5,6,7]. SLOX-1 is increased in vascular carotid plaque in patients with ischemic stroke [8]. The serum sLOX-1 level has been observed to be higher in patients with ACS compared to controls. Peak values of sLOX-1 in ACS were observed earlier than those of troponin T (TnT) [9]. However, a higher concentration of sLOX-1 in CAD patients after the PCI has not been completely investigated. The current study compares the utility of sLOX-1 levels in the risk assessment of patients of ACS/CAD for future recurrence. Peripheral blood was collected from the patients immediately after an emergent or scheduled coronary angiogram (CAG) and 24 h after PCI. If patients undergo PCI during an emergency, the sLOX-1 level has diagnostic potential and predicts ACS recurrence and death. Further in this study, the post-interventional sLOX-1 level was significantly high in recurrence. Thus, to the best of our knowledge, the evidence of increased sLOX-1 in recurrence cases could be information that is added to the existing understanding.

## 2. Materials and Methods

### 2.1. Study Population

Study subjects were recruited between May 2018 and June 2019 from the Lari Cardiology Centre, Department of Cardiology, King George Medical University, Lucknow, UP, India. All the cases that were recruited during this study period were included to constitute the sample size. Patients undergoing angiography and diagnosed with ACS and stable CAD (pre-PCI and post PCI) were included in the study. All angiographically proven subjects showing variable degrees of cardiac vessel blockage were categorized as cases, and the rest of the subjects as well as first degree relatives of these patients from the healthy population were categorized as control, and both of the groups were evaluated further by biochemical and hematological examination and follow-up for one year. All the patients who fulfilled the inclusion criteria were recruited for the study. Written informed consent was taken from the patients. The research related to human use has been complied with institutional policies and follows the tenets of the Declaration of Helsinki. The study was approved by the Institutional Ethical Committee of King George’s Medical University, Lucknow (ID 95th ECM II B-T/P13). Informed consent was obtained from all subjects involved in the study.

### 2.2. Sample Collection and Serum Isolation

A total of 3.5 mL of peripheral blood was collected from cases and control subjects in plain and EDTA vials (NOVAC, Polymed, Poly Medicure Ltd., Delhi, India). The serum was separated by centrifugation at 1900× *g* for 10 min and stored at −80 °C until further processing.

### 2.3. Biochemical Examination

All biochemical parameters, including serum thyroid stimulating hormone (TSH), 25-hydroxy vitamin D, vitamin B12, folate, and homocysteine (HCY), were measured by a fully automated biochemical analyzer (ARCHITECT i2000SR, Abbott Diagnostic, Chicago, IL, USA, and Selectra ProXL, ELITech Group, Puteaux, France). Selectra 500 Pro XL was used to measure magnesium and creatine kinase-MB (CK-MB) levels according to the manufacturer’s instructions. The neutrophil lymphocyte ratio (NLR) was calculated by taking the absolute count of neutrophils divided by the total count of lymphocytes. The platelet lymphocyte ratio (PLR) was calculated as the absolute count of platelets divided by the absolute count of lymphocytes. Complete blood count investigations involved measuring all differential values on Swelab Alfa Automated Hematology Analyzer (Boule Medical, Stockholm, Sweden).

### 2.4. ELISA for sLOX-1

The serum was separated by centrifugation (1900 g for 10 min) and stored at −80 °C for the quantification of sLOX-1 concentrations. The serum sLOX-1 was quantified by a commercially available enzyme-linked immunosorbent assay (ELISA) kit as per the manufacturer’s instructions (USCN, Wuhan, China). The kit was with an intra-assay coefficient of variation of <10% and an interassay CV of <12%. ELISA routinely analyzed all the samples in triplicate, and the results were averaged to minimize measurement errors and calculate the final concentration in samples. The level of the sLOX-1 level was measured in terms of ng/mL. We compared the diagnostic value of the sLOX-1 level of the different groups vs. the control group.

### 2.5. Follow-Up

After a primary PCI, all patients were monitored for at least 24 h. The patients were given standard medications by consulting physicians according to the up-to-date guidelines. After discharge, all patients were followed up for one year in an outpatient setting. Electronic patient records identified the recurrence of all patients. The follow-up started on the day of admission, and the patients were prospectively followed for 1 year with a standard medical treatment. All the patients were followed up completely. The recurrence of CAD was associated with recurrent cardiovascular events, including stroke and recurrent myocardial infarction (MI).

We analyzed the sLOX-1 as a diagnostic indicator across the spectrum of acute coronary syndrome, but a number of tables are related with recurrence or treatment outcome scenarios.

### 2.6. Statistical Analysis

The statistical analysis was performed using SPSS (statistical package for social sciences, IBM, Armonk, NY, USA) Version 21.0 statistical analysis software. Normally distributed continuous variables were presented as mean ±SD (standard deviation) and in number (%). The ANOVA test followed by post-hoc tests (Tukey’s HSD) was used to compare the within-group and between-group variances. A receiver operating characteristic curve (ROC) was developed to evaluate the diagnostic performance of sLOX-1. 2 × 2 tables built categorizing tests as positive or negative based on individual cut-offs obtained from ROC curves. A *p*-value < 0.05 (two-tailed) was considered significant.

## 3. Results

A total of 160 subjects, including 132 cases and 28 controls, were included in the study. The study subjects were divided into 4 groups: group I: patients underwent coronary angiography (*n* = 18), who underwent coronary angiography, but did not have established CAD; group II: patients of stable CAD underwent percutaneous intervention (*n* = 50); group III: patients of the acute coronary syndrome (*n* = 64); and group IV: healthy controls (*n* = 28). The age of study subjects ranged from 23 to 85 years of age. The mean age of patients enrolled in the study was 47.94 ± 14.30 years. The mean age of group I was 49.33 ± 15.66 years, that of group II was 46.40 ± 14.82 years, that of group III was 48.78 ± 14.36 years, and that of group IV was 47.89 ± 12.76 years. In the current study, the baseline characteristics, including age, gender, diabetes mellitus, and lipid profiles, were comparable and did not differ significantly between the groups. We aim to investigate and compare the pre/post PCI levels of sLOX-1 and other biochemical markers in stable ACS/CAD patients, followed up to one year to assess the future recurrence of the disease, and the sLOX-1 and other biochemical markers were measured in the predictor of recurrence of ACS/CAD.

### 3.1. Pre-Treatment Level of Serological Parameters in Study Groups

The pre-treatment serological parameters (neutrophil–lymphocyte ratio, platelet–lymphocyte ratio, vitamin D, magnesium level, CK-MB, HCY, sLOX-1, vitamin B12, TSH, and folate level) for all the study groups were compared and depicted in Table 1. The platelet–lymphocyte ratio was significantly lower in group IV than in group II and III (*p* < 0.001). Similarly, the vitamin D level was significantly higher in the control group (group IV with 31.84 ± 6.44 ng/mL), and group II was 16.29 ± 3.99 ng/mL, when compared to group I and III (*p* < 0.001). The CK-MB levels in group II patients (67.78 ± 73.22 IU/L) were significantly higher than group I, group III, and controls (group IV). The HCY level of group II (41.75 ± 45.10 μmol/L) was significantly higher than the levels of the other groups. For the groups least followed during the pre-treatment, the sLOX-1 level of group IV was 28.68 ± 9.97 units, whilst that of groups I, II, and III was 44.43 ± 42.86 units, 815.04 ± 683.38 units, and 995.16 ± 611.77 units, respectively (*p* < 0.001). The sLOX-1 levels of group I and group IV were comparable, while group II and group III had significantly higher sLOX-1 levels than group IV.

The comparisons of pre-treatment serological parameters between groups are shown in Table 2. The pre-treatment Vitamin D, CK-MB, and HCY level were significantly different between group I vs. group II, group II vs. group III, and group II vs. group IV (*p* < 0.001). The pre-treatment sLOX-1 level was significantly higher between group I vs. group II, group I vs. group III, and group II vs. group IV (*p* < 0.001). The folate level was significantly different between group I vs. group IV and group II vs. group IV (*p* < 0.001). The analysis of the group differences reveals that the CK-MB level was significantly different between group I vs. group II, group II vs. group III, and group II vs. group IV (*p* < 0.001).

### 3.2. Post-Interventional Serological Parameters

The post-intervention serological parameters in the different groups are depicted in Table 3. The serological parameters were compared amongst cases and controls: a significant difference was observed in the level of vitamin D in group II (16.25 ± 4.03 ng/mL, *p* < 0.001), CK-MB levels in group II (99.32 ± 84.80 IU/L, *p* < 0.001), and HCY levels in group II (61.18 ± 52.23 μmol/L, *p* < 0.001, *p* < 0.001); and a subsequent rise in the post-intervention sLOX-1 level of groups I, II, and III (74.01 ± 58.35 units, 1137.36 ± 772.59 units, and 1945.50 ± 843.90 units, respectively; *p* < 0.001) was observed. The maximum change in pre-treatment sLOX-1 levels was observed for group III (95.50%), followed by that in group I (66.57%); in group II, the change was kept to a minimum (39.55%) while rest parameters did not differ significantly.

The comparisons of post-interventional serological parameters between groups are shown in Table 4. The serological parameters of all the patients with different types of intervention (group I—coronary angiography; group II—stable CAD percutaneous intervention; and group III—acute CAD percutaneous intervention) were assessed. Comparing the post-intervention serological parameters of these patients, we found significant differences in the vitamin D, CK-MB, HCY, sLOX-1, and folate levels (*p* < 0.001).

### 3.3. Diagnostic Value of Pre-Treatment sLOX-1

**Figure 1:**
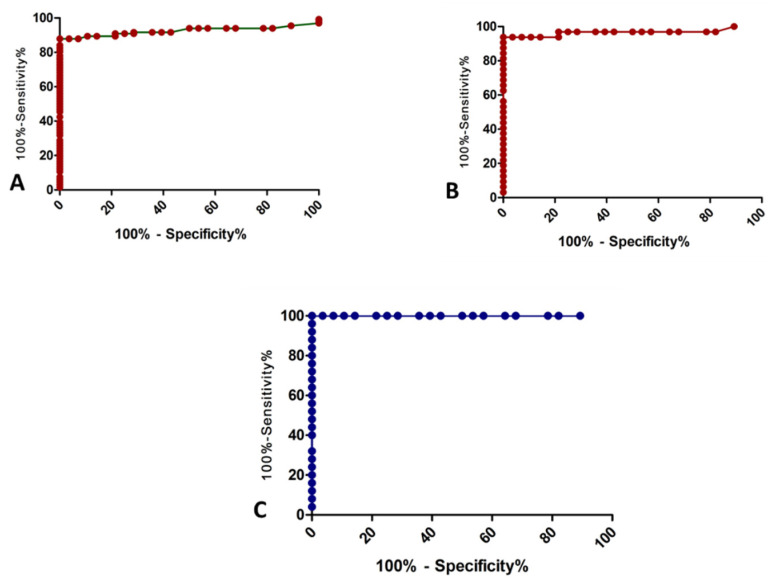
ROC curve analysis for (**A**) cases (groups I + II + III) vs. control, (**B**) ACS vs. control, and (**C**) stable CAD that underwent PCI vs. control.

A pre-treatment sLOX-1 ROC curve analysis was performed at a cut-off with a higher value that indicated the need for coronary angiography (Table 5). The area under the curve for sLOX-1 was 0.925 for cases that were discriminated from controls with a sensitivity and specificity of 87.88 and 100%, respectively. sLOX-1 showed 100% sensitivity and specificity in the discrimination of stable CAD that underwent PCI vs. controls with an AUC of 1.00 (Figure 1). The post-treatment sLOX-1 level was not included in the ROC curve analysis.

### 3.4. Recurrence of Coronary Artery Disease in Follow-Up Cases

The recurrence of CAD was associated with recurrent cardiovascular events, including stroke and recurrent myocardial infarction (MI). A recurrence of coronary artery disease was observed in 9 out of 132 (6.8%) cases. A recurrence of coronary artery disease was observed in 4.0% of group II and 10.9% of group III; however, no recurrence was observed in group I. The recurrence rate of CAD among the three groups was not statistically significant, as depicted in Table 6. The association of pre-treatment and post-interventional serological variables with recurrence was assessed and is depicted in Table 7. In recurrence cases, the pre-treatment sLOX-1 level was higher than no recurrence cases; however, the difference was not significant (*p* = 0.655). However, the post-interventional sLOX-1 level was significantly different and higher in recurrence cases (*p* = 0.027). Other pre- and post-interventional serological parameters did not show any association with recurrence.

## 4. Discussion

The current study compares the utility of sLOX-1 levels and other biochemical markers in the risk assessment of patients of acute coronary syndromes/CAD for future recurrence/morbidity/mortality. Our study showed a statistically significant increase in the pre-treatment levels of sLOX-1 in all the studied groups, with the order of increase being: patients with ACS > patients with stable CAD > patients who only underwent coronary angiography > controls. There was a statistically significant rise in the post-treatment sLOX-1 levels in all the patient groups except controls, with the order of increase being: patients with ACS > patients with stable CAD > patients who only underwent coronary angiography.

We have not performed a multivariate analysis and primarily focused on the independent use of the sLOX-1 level for prediction. We plan to perform a multivariate assessment in a separate study to see the association of sLOX-1 levels with different clinical and demographic conditions. Such multivariate assessment is out of the context of the present study and beyond its scope. Given the high sensitivity and specificity of the sLOX-1 level for predicting various coronary artery conditions, a multivariate assessment was not considered essential.

In coherence with our results, Hayashida et al. found sLOX-1 as a useful marker for the early diagnosis of ACS [9]. A study by Zhang et al. found a significant association between high sLOX-1 levels with the presence and severity of CAD in patients with metabolic syndrome [10]. Similarly, our study showed a higher sLOX-1 level in patients with ACS than in patients with stable CAD. Zhao et al. found a significant correlation of sLOX-1 levels with complex coronary vessel lesions that might help in the prediction of vulnerable plaques [11]. The study of Li et al. reported that the post-procedural serum sLOX-1 levels were significantly associated with the risk of in-stent restenosis (ISR) and with the severity of lumen loss in patients with stable coronary artery disease undergoing primary PCI. Post-procedural serum sLOX-1 levels might be a useful marker for the detection and risk assessment of ISR after PCI [12]. We have also tried to test whether post-procedural serum sLOX-1 levels might be useful for the detection and risk assessment of a further recurrence after PCI. However, we did not find any significant association in post-interventional sLOX-1. In ACS patients undergoing emergency PCI, the sLOX-1 level has been found to have diagnostic potential and predict ACS recurrence or death [13]. In our study, the post-interventional sLOX-1 level was significantly higher in recurrence cases (*p* = 0.027). In the present study, pre-treatment CK-MB levels in stable CAD patients were statistically higher than those in other groups. Post-interventional increases in CK-MB levels were found to be statistically significant for all three patient groups. A study by Ullah et al. reported that elevated cardiac biomarkers after PCI might be associated with worse clinical outcomes. This also helps to identify high-risk post PCI groups [14]. However, in our study, the difference in the recurrence rate of CAD among the above three groups did not correlate with an increase in the pre-treatment and post-treatment CK-MB levels.

In our study, pre-treatment HCY levels in stable CAD patients were significantly higher than those in other groups. A post-treatment increase in HCY levels was found to be statistically significant for all three patient groups. In a study by Rajabi et al., the mean serum homocysteine of diabetic patients (type II) with CAD was higher than those with normal coronary arteries and was significantly higher in patients with three-vessel involvement than those with normal coronary arteries that had no vessel involvement [15]. We have also found significantly higher serum homocysteine levels in stable CAD and ACS patients. Kurtul et al. found that the platelet–lymphocyte ratio (PLR) was significantly associated with the severity and complexity of coronary atherosclerosis in patients with ACS [16]. We did not find any association of the PLR between stable CAD and ACS. In our study, a change in pre-treatment neutrophil–lymphocyte ratio (NLR) was highest in group II (36.23%), followed by group III (34.16%), while in group I, the change was kept to a minimum (27.56%). The NLR was significantly higher in patients with stable CAD > patients with ACS > control patients. However, there was no statistical significance in the post-interventional levels of NLR. Arbel et al. found that neutrophil/lymphocyte ratio was independently associated with CAD severity and 3-year outcome. The NLR value appears adjunct to conventional risk factors, and commonly used biomarkers [17]. We did not find any NLR value association in predicting recurrence or death.

There were no significant differences in serum magnesium levels in intergroup comparison of pre- or post-treatment levels. Qin et al. reported that serum levels of vitamin B12, vitamin D, and vitamin C were in close correlation to the vascular endothelial dysfunction in CAD patients [18]. In our study, we did not find any association of vitamin B12 with CAD. Similarly, a study by Kim et al. did not find any association of serum folate and vitamin B12 with the increased risk of atherosclerotic events [19]. The findings of our study suggested that there might be a correlation between lower folate levels and CAD/ACS in Indian patients. In our study, the patients who only underwent coronary angiography and patients with stable CAD and ACS had lower vitamin D levels than controls. However, this could be attributed to the lack of sun exposure as the morbid patients are kept indoors, which may be a coincidental finding. Studies have reported the homocysteine level and vitamin B12 may be an independent risk factor for coronary artery disease [20,21].

## 5. Conclusions and Limitations

We have comparatively analyzed the sLOX-1 level as an independent diagnostic marker of ACS with few established markers and tried to assess whether its levels before and after PCI may predict the risk of a future recurrence. Consistent with the earlier studies, we have found increased sLOX-1 levels in all the patient groups with a statistically significant increase in all the groups before and after PCI. We have also found a significant correlation of post-treatment sLOX-1 level with a future recurrence. The level of sLOX-1 is associated with the incidence of periprocedural myocardial infarction [7]. Our findings suggest that circulating levels of sLOX-1 might be a diagnostic and prognostic marker for atherosclerotic-related events. In our study, sLOX-1 is a more sensitive and specific biomarker for ACS that provides additional diagnostic values; however, we have not compared this with TnT. This finding is also supported by other studies [13,21,22,23].

In our study, nutritional and hormonal status showed inconsistent results. There was no significant increase or decrease in these entities’ pre- or post-PCI levels, except for low folate levels, which showed significant association with CAD/ACS, thus warranting further investigation in larger studies from an Indian perspective. Considering the very short duration of this study, it is recommended to conduct a multicentric study with long-term follow-up to achieve a better outcome. For instance, this study has limitations of a small-sized observational study. Risk factors, including smoking, diabetes, and body mass index (BMI), which were different in the groups, were not analyzed and may have affected the results of the biomarkers studied.

## Figures and Tables

**Table 1:** Intergroup comparison of pre-treatment serological parameters.

	Group I (*n* = 18)	Group II (*n* = 50)	Group III (*n* = 64)	Group IV (*n* = 28)	ANOVA	
	Mean ± SD	Mean ± SD	Mean ± SD	Mean ± SD	F Value	*p* Value
Neutrophil–Lymphocyte Ratio	1.22 ± 0.677	1.90± 1.428	1.88 ± 1.377	1.45 ± 0.86	2.029	0.112
Platelet–Lymphocyte Ratio	48.57 ± 24.33	52.60 ± 25.20	52.13 ± 24.89	30.38 ± 13.67	6.614	<0.001
Vitamin D	10.76 ± 5.08	16.29 ± 3.99	10.16 ± 4.52	31.84 ± 6.44	139.232	<0.001
Magnesium	1.45 ± 0.303	1.344 ± 0.369	1.52 ± 0.339	1.56 ± 0.516	2.753	0.045
Creatinine Kinase-MB	17.39 ± 26.35	67.78 ± 73.22	14.78 ± 19.97	14.96 ± 3.82	16.456	<0.001
Homocysteine	5.31 ± 2.77	41.75 ± 45.10	9.51 ± 12.15	9.92 ± 2.50	17.762	<0.001
Vitamin B12	323.61 ± 230.48	282.60 ± 207.82	306.56 ± 453.74	340.29 ± 260.14	0.192	0.902
Thyroid Stimulating Hormone	3.83 ± 4.76	3.73 ± 4.15	3.08 ± 4.22	2.68 ± 0.99	0.612	0.608
Folate	13.62 ± 5.44	14.72 ± 5.78	10.73 ± 9.02	23.70 ± 10.27	16.948	<0.001
sLOX-1	44.43 ± 42.86	815.04 ± 683.38	995.16 ± 611.77	28.68 ± 9.97	29.492	<0.001

**Table 2:** Comparison of the pre-treatment serological parameters between the groups.

Serological Parameters	Group I vs. Group II			Group I vs. Group III			Group II vs. Group III			Group I vs. Group IV			Group II vs. Group IV		
	Mn Diff.	SE	*p* Value	Mn Diff.	SE	*p* Value	Mn Diff.	SE	*p* Value	Mn Diff.	SE	*p* Value	Mn Diff.	SE	*p* Value
Neutrophil–Lymphocyte Ratio	−0.67	0.35	0.214	−0.66	0.34	0.206	0.01	0.24	1.000	−0.23	0.38	0.931	0.44	0.30	0.445
Platelet–Lymphocyte Ratio	−4.02	6.43	0.924	−3.55	6.24	0.941	0.47	4.41	1.000	18.19	7.06	0.053	22.22	5.52	0.001
Vitamin D	−5.53	1.32	<0.001	0.60	1.29	0.966	6.13	0.91	<0.001	−21.08	1.46	<0.001	−15.55	1.14	<0.001
Magnesium	0.11	0.10	0.695	−0.06	0.10	0.923	−0.18	0.07	0.067	−0.10	0.12	0.811	−0.22	0.09	0.079
Creatinine Kinase-MB	−50.39	12.05	<0.001	2.61	11.70	0.996	53.00	8.28	< 0.001	2.42	13.25	0.998	52.82	10.35	<0.001
Homocysteine	−36.44	7.28	<0.001	−4.20	7.06	0.934	32.24	5.00	<0.001	−4.61	8.00	0.939	31.83	6.25	<0.001
Vitamin B12	41.01	92.89	0.971	17.05	90.16	0.998	−23.96	63.79	0.982	−16.67	102.10	0.998	−57.69	79.77	0.888
Thyroid Stimulating Hormone	0.09	1.07	1.000	0.74	1.04	0.893	0.65	0.74	0.815	1.14	1.18	0.768	1.05	0.92	0.666
Folate	−1.10	2.21	0.959	2.88	2.15	0.537	3.99	1.52	0.047	−10.09	2.43	<0.001	−8.98	1.90	<0.001
sLOX-1	−770.61	150.06	<0.001	−950.72	145.65	<0.001	−180.12	103.04	0.303	15.75	164.93	1.000	786.36	128.86	<0.001

**Table 3:** Intergroup comparison of post-intervention serological parameters.

	Group I (*n* = 18)	Group II (*n* = 50)	Group III (*n* = 64)	ANOVA	
	Mean ± SD	Mean ± SD	Mean ± SD	F Value	*p* Value
Neutrophil–Lymphocyte Ratio	1.565 ± 0.63	2.588 ± 1.78	2.53 ± 1.67	2.915	0.058
Platelet–Lymphocyte Ratio	56.95 ± 28.26	65.95 ± 27.10	65.66 ± 27.06	0.821	0.442
Vitamin D	10.82 ± 5.16	16.25 ± 4.03	10.13 ± 4.53	28.262	<0.001
Magnesium	1.358 ± 0.570	1.494 ± 0.654	1.546 ± 0.641	0.616	0.542
CK-MB	34.00 ± 32.47	99.32 ± 84.80	30.25 ± 26.79	22.490	<0.001
Homocysteine	15.14 ± 5.48	61.18 ± 52.23	18.73 ± 16.49	24.825	<0.001
Vitamin B12	321.50 ± 230.74	282.96 ± 207.86	307.67 ± 453.61	0.107	0.898
Thyroid Stimulating Hormone	3.83 ± 4.77	3.74 ± 4.15	3.08 ± 4.22	0.428	0.653
Folate	13.85 ± 5.69	14.73 ± 5.84	10.87 ± 8.93	3.957	0.021
sLOX-1	74.01 ± 58.35	1137.36 ± 772.59	1945.50 ± 843.90	47.053	<0.001

**Table 4:** The comparison of post-interventional serological parameters between groups.

	Group I vs. Group II			Group I vs. Group III			Group II vs. Group III		
	Mn Diff.	SE	*p* Value	Mn Diff.	SE	*p* Value	Mn Diff.	SE	*p* Value
Neutrophil–Lymphocyte Ratio	−1.02	0.45	0.060	−0.97	0.43	0.070	0.06	0.31	0.981
Platelet–Lymphocyte Ratio	−9.00	7.49	0.454	−8.71	7.27	0.456	0.29	5.14	0.998
Vitamin D	−5.43	1.22	<0.001	0.69	1.18	0.828	6.12	0.84	<0.001
Magnesium	−0.14	0.18	0.719	−0.19	0.17	0.512	−0.05	0.12	0.901
CK-MB	−65.32	15.60	<0.001	3.75	15.14	0.967	69.07	10.71	<0.001
Homocysteine	−46.04	9.41	<0.001	−3.59	9.14	0.918	42.45	6.46	<0.001
Vitamin B12	38.54	96.76	0.916	13.83	93.92	0.988	−24.71	66.44	0.927
Thyroid Stimulating Hormone	0.09	1.17	0.997	0.75	1.14	0.786	0.66	0.81	0.691
Folate	−0.87	2.06	0.906	2.99	2.00	0.297	3.86	1.41	0.020
sLOX-1	−1063.35	208.43	<0.001	−1871.49	202.31	<0.001	−808.14	143.12	<0.001

**Table 5:** Diagnostic of sLOX-1 in CAD.

Diagnostic	Cut-Off Value	AUC	*p*-Value	Sensitivity (95% CI)	Specificity (95% CI)
Cases (groups I + II + III) vs. control	≥47.15	0.925	<0.0001	87.88 (81.06–92.91)	100.00 (87.66–100.0)
ACS vs. control	≥48.50	0.966	0.01	93.75 (84.76–98.27)	100.00 (87.66–100.0)
Stable CAD that underwent PCI vs. control	≤47.50	1.00	<0.0001	100.00 (87.66–100.0)	100.00 (92.89–100.0)

**Table 6:** Intergroup comparison of recurrence in the study population.

Recurrence	Total (*N* = 132)	Group I (*n* = 18)		Group II (*n* = 50)		Group III (*n* = 64)	
No recurrence	123	18	100	48	96	57	89.10
Recurrence	9	00	0.0	02	4.0	07	10.90

χ^2^ = 3.651; *p* = 0.161.

**Table 7:** Association of pre-treatment and post-interventional serological parameters with recurrence.

	No Recurrence (*n* = 123)	Recurrence (*n* = 9)		
	Mean ± SD	Mean ± SD	‘t’	*p*-Value
**(a) Pre-treatment**				
Neutrophil–Lymphocyte Ratio	1.83 ± 1.37	1.47 ± 0.59	0.776	0.439
Platelet–Lymphocyte Ratio	52.77 ± 25.13	38.77 ± 14.68	1.647	0.102
Vitamin D	12.63 ± 5.20	11.68 ± 6.37	0.522	0.603
Magnesium	1.43 ± 0.36	1.65 ± 0.16	−1.839	0.068
Creatinine Kinase-MB	36.59 ± 55.71	16.33 ± 19.39	1.083	0.281
Homocysteine	21.90 ± 34.05	10.84 ± 11.51	0.968	0.335
Vitamin B12	300.81 ± 351.39	286.11 ± 344.92	0.121	0.904
Thyroid Stimulating Hormone	3.45 ± 4.18	3.23 ± 5.40	0.147	0.884
Folate	12.50 ± 7.72	14.50 ± 7.28	−0.754	0.452
sLOX-1	790.18 ± 670.93	894.44 ± 725.25	−0.448	0.655
**(b) Post-interventional**				
Neutrophil–Lymphocyte Ratio	2.45 ± 1.70	1.97 ± 0.58	0.853	0.395
Platelet–Lymphocyte Ratio	65.76 ± 27.48	48.46 ± 16.80	1.860	0.065
Vitamin D	12.60 ± 5.22	11.80 ± 6.32	0.436	0.664
Magnesium	1.47 ± 0.63	1.87 ± 0.60	−1.822	0.071
CK-MB	58.69 ± 67.09	32.78 ± 26.29	1.149	0.253
Homocysteine	35.35 ± 41.05	20.34 ± 16.24	1.087	0.279
Vitamin B12	301.07 ± 351.29	288.22 ± 345.58	0.106	0.916
Thyroid Stimulating Hormone	3.45 ± 4.18	3.19 ± 5.39	0.170	0.865
Folate	12.58 ± 7.69	14.81 ± 7.45	−0.841	0.402
sLOX-1	1332.81 ± 967.50	2086.22 ± 1079.43	−2.238	0.027

## Data Availability

Not applicable.
